# Literary Notes

**Published:** 1910-11

**Authors:** 


### LITERARY NOTES

Rebman Company, medical publishers, 1123 Broadway, New York, have just issued a new catalogue comprising a number of important books of recent issue. It is handsomely printed on well finished tinted paper and is completely indexed. It is accompanied by a list of art prints for the waiting room and home of the doctor. It will be furnished on application.

F. A. Davis Company, Philadelphia, have recently sent out a handsome catalague of medical works, which they publish in the most approved form. It contains numerous medallion portraits of the authors of the books they publish, is well indexed and is a convenient brochure of reference. For copies, address the publishers.

